# Rate of Occult Cervical Lymph Node Involvement in Supraglottic Squamous Cell Carcinoma

**Published:** 2017-05

**Authors:** Maziar Motiee Langroudi, Behrooz Amirzargar, Amin Amali, Mohammad Sadeghi, Mehrdad Jafar, Mohammad Reza Hoseini, Fatemeh Tavakolnejad

**Affiliations:** 1 *Otorhinolaryngology Research Center, Tehran University of Medical Sciences, Tehran, Iran.*

**Keywords:** Laryngeal cancer, Lymph nodes, Metastasis, Neck dissection, Squamous cell carcinoma

## Abstract

**Introduction:**

To assess the rate of cervical lymph node involvement in patients with supraglottic squamous cell carcinoma (SCC) with no lymph node in clinical assessments and radiological studies.

**Materials and Methods:**

Fifty-six patients who underwent elective dissection of the cervical lymph node of the second through fourth level were enrolled, and pathologic evaluation of the dissected lymph nodes was performed. Lymph node involvement and association between tumor grade, smoking and gender with lymph node involvement were assessed.

**Results:**

The rate of the occult neck metastasis in this series was 37.5%. There was no statistically significant association between lymph node involvement and tumor grade, smoking, or gender.

**Conclusion:**

Based on the results of our study, we recommend elective bilateral neck dissection in all stages of N0 supraglottic SCC patients.

## Introduction

Squamous cell carcinoma (SCC) is the most common cancer of the larynx and accounts for 90% of malignant laryngeal tumors. Supraglottic SCC after glottic SCC is the second most common malignant tumor of the larynx (1,2). The male-to-female (M/F) ratio is 3.8:1 for all laryngeal cancers, and more than 90% of cancers occur in patients over 40 years of age (3).

The two most important risk factors for all laryngeal cancers are smoking and alcohol consumption, and more than 97% of patients have a history of cigarette smoking (1,4). Other risk factors include laryngopharyngeal reflux, human papillomavirus (HPV), and toxins. Because of its rich network of lymphatics, supraglottic SCC has the most cervical lymph node metastasis among laryngeal cancers, and usually metastasizes to cervical lymph node levels II, III, and IV (5). The rate of pathologically involved lymph nodes in patients with supraglottic SCC who have no lymph node involvement in clinical assessments and radiological studies (occult lymph node metastasis) differs across studies and varies from 12% to 40%. On the other hand, the most important prognostic factor of supraglottic SCC is the presence of lymphatic metastasis, and 5-year survival rate decreases to 50% when there is lymphatic metastasis (6–8).

The classic approach to supraglottic SCC with no clinical or radiological cervical lymph node metastasis (N0) is surgical treatment with bilateral neck dissection of levels II, III, and IV. If the risk of occult cervical lymph node metastasis is statistically more than 20%, lymph node dissection is necessary because neck dissection in less than 20% metastasis imposes potential complications such as nerve and vessel injuries and bleeding and increases the time of surgery.

Several studies reported different rates of occult cervical lymph node metastasis (9–11), and some of these studies suggest that there was no need for neck dissection in patients with T1 N0 supraglottic SCC. The aim of this study was to assess the rate of occult cervical lymph node metastasis in N0 supraglottic SCC in the Middle East population, especially in the lower stages of laryngeal cancer, in order to potentially prevent unnecessary neck dissection in patients referred for supraglottic SCC treatment.

## Materials and Methods

This was a retrospective study. Patients undergoing level II through IV elective neck dissection for supraglottic SCC between 2002 and 2014 were enrolled. The study was conducted at the tertiary center of Imam Khomeini Hospital Complex, Valiasr Hospital at Tehran University of Medical Sciences. All patients were classified as N0, meaning that they had no cervical lymph node metastasis in the clinical and radiological evaluation.

After meeting the inclusion criteria, 56 patients were enrolled. Histopathological examination of all samples was performed at the Cancer Institute of Imam Khomeini complex. Grading of tumors was based on direct laryngoscopy and biopsy, imaging and pathologic reports (T1–T4). Exclusion criteria were incomplete patient profile and the pathology reports from other centers. The associations between lymph node involvement and tumor grade, smoking, and gender were also assessed.

The IBM SPSS statistics package version 22 was used for analysis. A Chi-square test was used for the analysis of the associations between qualitative variables.

The study was approved by the Ethics Committee of the Tehran University of Medical Sciences and the National Medical Ethics Committee according to the principles of the Declaration of Helsinki.

## Results

The mean age of patients was 57.6 ± 11 years. The M/F ratio was 27:1 (54:2). There was no statistically significant association between cervical lymph node metastasis and gender (chi-square test [n=56]; p=0.710).

Patients were classified into four groups based on the amount of cigarette smoking per year (less than 20 pack year [p/y], 20–40 p/y, 40–60 p/y, and more than 60 p/y). Most patients (42.9%) were in 40–60 p/y group. There was no statistically significant association between histopathological involvement and cigarette smoking (chi-square test [n=56]; p=0.854). There was no history of alcohol consumption in our patients.Twenty-one of 56 patients had cervical lymph node involvement based on histopathologic evaluation, so the rate of occult cervical metastasis was 37.5% in this series. Ten of 21 patients (47.6%) with cervical lymph node metastasis had bilateral neck involvement.

Cervical lymph node metastasis based on tumor grade is summarized in Table 1. Most patients were in the T3 group (44.6%). The association between tumor grade and lymph node involvement was assessed and showed no statistically significant association between tumor grade and cervical lymph node metastasis (chi-square test [n=56]; P=0.389).

**Table 1:** Cervical lymph node metastasis base on the tumor grade

	**Tumor Grade**	**Lymph Node Involvement**		**Total**	**Percentage**
		**Yes** (%)	**No** (%)		
	T1	1 (16.7)	5 (83.3)	6	10.7
	T2	4 (28.6)	10 (71.4)	14	25.0
	T3	10 (40)	15 (60)	25	44.6
	T4	6 (54.5)	5 (45.5)	11	19.6
Total		21	35	56	100

## Discussion

Our study demonstrated a significant rate of cervical lymph node metastasis in patients with supraglottic SCC. Several articles have previously reported different rates of cervical lymph node metastasis, from 12% to 40% (6–11).

Despite the classic approach to patients with N0 supraglottic SCC, which include bilateral neck dissection of levels II through IV, some authors believe that treatment of the neck in the patients with T1 N0 supraglottic SCC is not necessary (11).

Redaelli et al. showed that the risk of occult cervical metastasis in T1 patients was significantly lower than other tumor grades (11), but earlier studies showed no relation between tumor grade and cervical metastasis (12). In our study the rate of cervical nodal metastasis varied from 16.7% to 54.5% in T1 and T4 patients, respectively, but there was no statistically significant association between tumor grade and cervical lymph node metastasis. This, therefore, emphasizes the role of neck dissection for all stages.

One of the important findings in our study was that 47.6% of patients had bilateral cervical lymph node metastasis. This finding contrasts with other studies that recommended unilateral neck dissection (13).

Different studies have reported different M/F ratios. However, in our series, M/F was 27:1, which is greater than in other studies. This is potentially because of religious factors in our country, and because a large percentage of smokers are men, especially at older ages.

There were no patients with a history of alcohol consumption in our series, although it is an important risk factor for laryngeal SCC and a more important risk factor for supraglottic SCC. Once again, this is because of religious factors in our society.

One limitation of this study is the low sample size, which may affect the results. Further studies with more patients could provide us with more accurate conclusions.

## Conclusion

This study demonstrated a significant rate of cervical lymph node metastasis in patients with supraglottic SCC. We therefore recommend elective bilateral neck dissection in all stages of N0 supraglottic SCC patients.

## References

[1] De Stefani E (2004). Supraglottic and glottic carcinomas: epidemiologically distinct entities?. Int J Cancer Journal.

[2] Hoffman HT (2006). Laryngeal cancer in the United States: changes in demographics, patterns of care, and survival. The Laryngoscope.

[3] Jemal A, Siegel R, Ward E, Murray T, Xu J, Thun MJ (2007). Cancer statistics, 2007. CA Cancer J Clin.

[4] Menvielle G, Luce D, Goldberg P, Bugel I, Leclerc A (2004). Smoking, alcohol drinking and cancer risk for various sites of the larynx and hypopharynx A case-control study in France. Eur J Cancer Prevent.

[5] Candela FC, Shah J, Jaques DP, Shah JP (1990). Patterns of cervical node metastases from squamous carcinoma of the larynx. Arch Otolaryngol Head Neck Surg.

[6] Esposito ED, Motta S, Cassiano B, Motta G (2001). Occult lymph node metastases in supraglottic cancers of the larynx. Otolaryngol Head Neck Surg.

[7] Gallo O, Fini-Storchi I, Napolitano L (2000). Treatment of the contralateral negative neck in supraglottic cancer patients with unilateral node metastases (N1-3). Head Neck.

[8] Spriano G, Piantanida R, Pellini R, Muscatello L (2003). Elective treatment of the neck in squamous cell carcinoma of the larynx: clinical experience. Head Neck.

[9] Deganello A, Gitti G, Meccariello G, Parrinello G, Mannelli G, Gallo O (2011). Effectiveness and pitfalls of elective neck dissection in N0 laryngeal cancer. Acta Otorhinolaryngol Ital.

[10] Hicks WL (1999). Patterns of nodal metastasis and surgical management of the neck in supraglottic laryngeal carcinoma. Otolaryngol Head Neck Surg.

[11] Redaelli de Zinis LO (2002). The distribution of lymph node metastases in supraglottic squamous cell carcinoma: therapeutic implications. Head Neck.

[12] Ali S, Tiwari RM, Snow GB (1985). False-positive and false-negative neck nodes. Head Neck Surg.

[13] Cagli S, Yuce I, Yigitbasi OG, Güney E (2007). Is routine bilateral neck dissection absolutely necessary in the management of N0 neck in patients with supraglottic carcinoma?. Eur Arch Otorhinolaryngol.

